# Detection of Lymph Node Metastases in Human Colorectal Cancer by Using 5-Aminolevulinic Acid-Induced Protoporphyrin IX Fluorescence with Spectral Unmixing

**DOI:** 10.3390/ijms141123140

**Published:** 2013-11-21

**Authors:** Kenichi Harada, Yoshinori Harada, Masatomo Beika, Noriaki Koizumi, Koji Inoue, Yasutoshi Murayama, Yoshiaki Kuriu, Masayoshi Nakanishi, Takeo Minamikawa, Yoshihisa Yamaoka, Ping Dai, Akio Yanagisawa, Eigo Otsuji, Tetsuro Takamatsu

**Affiliations:** 1 Department of Pathology and Cell Regulation, Graduate School of Medical Science, Kyoto Prefectural University of Medicine, 465 Kajii-cho Hirokoji Kawaramachi, Kamigyo-ku, Kyoto 602-8566, Japan; E-Mails: daisuke@koto.kpu-m.ac.jp (K.H.); beika@koto.kpu-m.ac.jp (M.B.); nkoizumi@koto.kpu-m.ac.jp (N.K.); tminami@koto.kpu-m.ac.jp (T.M.); yamaoka@koto.kpu-m.ac.jp (Y.Y.); dping@koto.kpu-m.ac.jp (P.D.); ttakam@koto.kpu-m.ac.jp (T.T.); 2 Division of Digestive Surgery, Department of Surgery, Graduate School of Medical Science, Kyoto Prefectural University of Medicine, 465 Kajii-cho Hirokoji Kawaramachi, Kamigyo-ku, Kyoto 602-8566, Japan; E-Mails: i-koji@koto.kpu-m.ac.jp (K.I.); murayama@koto.kpu-m.ac.jp (Y.M.); kuriu@koto.kpu-m.ac.jp (Y.K.); mnakan@koto.kpu-m.ac.jp (M.N.); otsuji@koto.kpu-m.ac.jp (E.O.); 3 Department of Surgical Pathology, Graduate School of Medical Science, Kyoto Prefectural University of Medicine, 465 Kajii-cho Hirokoji Kawaramachi, Kamigyo-ku, Kyoto 602-8566, Japan; E-Mail: yanagisa@koto.kpu-m.ac.jp

**Keywords:** colorectal cancer, 5-aminolevulinic acid, lymph node metastasis, spectral unmixing, protoporphyrin IX

## Abstract

Accurate evaluation of metastatic lymph nodes (LNs) is indispensable for adequate treatment of colorectal cancer (CRC) patients. Here, we demonstrate detection of metastases of human CRC in removed fresh LNs using 5-aminolevulinic acid (ALA)-induced protoporphyrin IX (PpIX) fluorescence. A spectral unmixing method was employed to reduce the overlap of collagen autofluorescence on PpIX fluorescence. A total of 17 surgery patients with advanced CRC were included in this study. After 5-ALA at a dose of 15 mg/kg of body weight was applied orally 2 h prior to surgery, 87 LNs were subjected to spectral fluorescence imaging and histopathological diagnosis, and statistical analysis was performed. No apparent side effect was observed to be associated with 5-ALA administration. The spectral unmixing fluorescence intensity of PpIX in metastatic LNs was 10.2-fold greater than that in nonmetastaic LNs. The receiver-operating-characteristic (ROC) analysis showed that the area under the curve (AUC) was calculated as 0.95. Our results show the potential of 5-ALA-induced PpIX fluorescence processed by spectral unmixing for detecting metastases in excised fresh LNs from patients with CRC, suggesting that this rapid and feasible method is applicable to gross evaluation of resected LN samples in pathology laboratories.

## 1. Introduction

Colorectal cancer (CRC) is the second most common cause of cancer mortality and the most frequent cancer in many countries. It is considered to be a disease associated with the Western lifestyle [1]. Lymph node (LN) metastasis is a feature associated with advanced CRC [2,3]. There is mounting evidence from large population-based studies that the number of LNs examined by pathologists has a significant impact on survival in CRC. Accurate diagnosis of surgically-excised LNs is necessary for evaluating patient prognoses and treating patients with adjuvant chemotherapy [4–8].

Among detection methods for LN metastases, histopathological analysis of LNs is regarded as a gold standard. However, routine histopathological analysis of LNs has been shown to have a limited sensitivity for the detection of CRC metastases [9–12]; in general, only a few hematoxylin-eosin (H & E)-stain slides from the excised LNs are analyzed without macroscopic identification in a routine work. It is reported that there is a significant risk of underestimating nodal metastasis in the routine pathological examinations [13,14]. Therefore, a new method for detecting metastatic foci of removed LNs is desirable.

Fluorescence diagnosis using 5-aminolevulinic acid (5-ALA) has been used to detect the extent of primary tumors such as brain and urinary bladder tumors. Fluorescence of protoporphyrin IX (PpIX) accumulated after 5-ALA administration can be used for selective detection of primary epithelial malignant tumors [15–17]. In noncancerous cells, fluorescent PpIX is rapidly metabolized to nonfluorescent heme. In contrast, PpIX emitting a red fluorescence around 635 nm on blue light excitation selectively accumulates in cancer cells due to a decreased activity of ferrochelatase and/or an increased activity of porphobilinogen deaminase [18–20].

We have previously reported that 5-ALA is a sensitive probe for detection of LN metastasis in a murine model of rectal cancer and that its diagnostic accuracy was high [21]. We have shown that this feasible diagnostic approach is applicable to gross identification of metastases of resected fresh node samples of mice. However, as far as we know, there has been no report on whether 5-ALA administration is also effective in detecting LN metastases in human CRCs. In this study, we sought to evaluate the potential of oral administration of 5-ALA for detection of metastatic foci in excised regional LNs of CRC patients. We have also previously revealed that PpIX fluorescence emitted from cancer cells is sometimes covered by strong collagen autofluorescence, resulting in false negative result, in LNs of gastric cancer patients [22]. Therefore, it is important to exclude the unfavorable influence of collagen autofluorescence in tissues to specifically detect PpIX. To avoid this phenomenon, we analyzed acquired fluorescence images by using a spectral unmixing method in this study [23]. The method is reported to be useful for resolving crosstalk in a multispectral fluorescence image, and has a potential to contribute to specific detection of PpIX fluorescence. The results showed that PpIX fluorescence signal processed by the spectral unmixing method was effective for detecting CRC metastases in excised fresh human LNs.

## 2. Results

### 2.1. Separation of Overlapping Fluorescence Signals Using Spectral Unmixing

**Figure 1:**
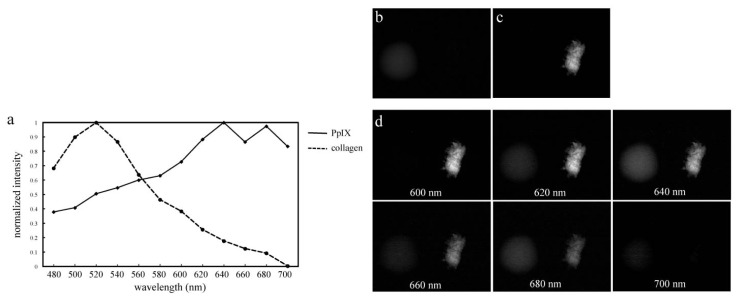
Spectra and spectroscopic images of protoporphyrin IX (PpIX) and collagen as references for spectral unmixing. Emission profiles of PpIX dissolved in dimethyl sulfoxide (DMSO) and of collagen powder (**a**) analyzed by the system; Acquired images of PpIX (**b**) and collagen (**c**) after spectral unmixing based on the reference spectra shown in (**a**); (**d**) Multispectral fluorescence images of PpIX (left side in each panel of (**d**)) and collagen (right side in each panel of (**d**)) acquired from 600 to 700 nm in 20-nm steps.

A preliminary experiment was performed in order to evaluate the spectral unmixing method employed in this study. Figure 1a shows fluorescence spectra of PpIX solution and collagen type I powder acquired by using the system. The fluorescence spectra of PpIX and collagen had characteristic peaks around 640 nm and 520 nm wavelengths, respectively. After acquisition of fluorescence spectroscopic images of PpIX solution and collagen powder from 480 to 700 nm in 20-nm steps using the system, spectral unmixed images of PpIX (Figure 1b) and collagen (Figure 1c) were created. The spectral unmixed images were created based on the reference spectra shown in Figure 1a. Fluorescence spectroscopic images of PpIX solution (left side in each panel of Figure 1d) and collagen powder (right side in each panel of Figure 1d) acquired from 600 to 700 nm in 20-nm steps are depicted for reference. PpIX solution mainly emitted fluorescence at the range from 620 nm to 680 nm and had highest signal intensity on the fluorescence image at 640 nm (Figure 1d). Collagen powder also strongly emitted fluorescence at the range from 600 nm to 680 nm. Thus, collagen fluorescence overlapped PpIX fluorescence as evaluated by using band-pass filtering. However, the fluorescence signal of PpIX was specifically separated from that of the collagen in the unmixed images, as shown in Figure 1b,c.

### 2.2. Enrolled Patients and LNs

Seventeen patients with CRC undergoing surgery with a total of 106 excised LNs were enrolled in this study. The average duration time of the surgeries was 4.05 ± 1.03 h. The average time between oral dose of 5-ALA and fluorescence imaging was 6.18 ± 1.03 h. Data from 19 LNs from three patients were eliminated because of the time limitation. In the eliminated three patients, the times between 5-ALA administration and completion of surgery were 4, 12.5 and 4 h, respectively.

After elimination of these data, the final study population consisted of nine men and five women with age ranges of 46 to 72 years and 61 to 83 years, respectively (Table 1). Of the 87 LNs, 32 were diagnosed as metastatic LNs, and 55 as non-metastatic LNs. No apparent side effect was observed in this study.

### 2.3. Imaging of Excised LNs in CRC Patients

**Figure 2:**
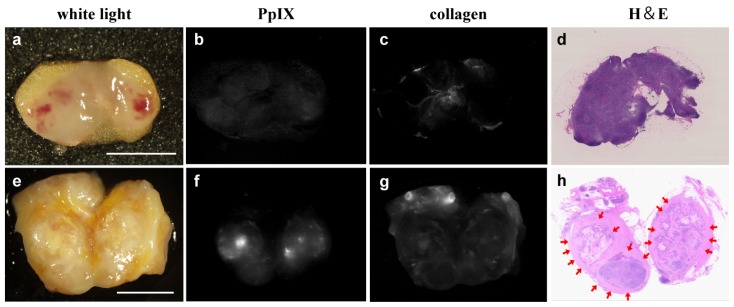
Imaging of a metastatic and a non-metastatic lymph node (LN) by using spectral unmixing. Images of a non-metastatic LN (**a**–**d**) and a metastatic LN (**e**–**h**) cut in half are shown; (**a**,**e**) White-light images; (**b**,**f**) Spectral unmixed fluorescent signal of PpIX; (**c**,**g**) Spectral unmixed fluorescent signal of collagen; (**d**,**h**) H & E-stained images. Red arrows indicate metastatic lesions. Scale bar = 5 mm.

All of the images were acquired by the first author. The representative images of a non-metastatic (Figure 2a–d) and a metastatic (Figure 2e–h) LNs cut in half are shown. Non-metastatic LN showed no apparent spectral unmixing PpIX fluorescence (Figure 2b). In contrast, spectral unmixed fluorescence signal of PpIX was clearly observed in metastatic lesions, as shown in Figure 2f.

### 2.4. Diagnostic Performance of 5-ALA-Induced PpIX by Using the Spectral Unmixing Method

**Figure 3:**
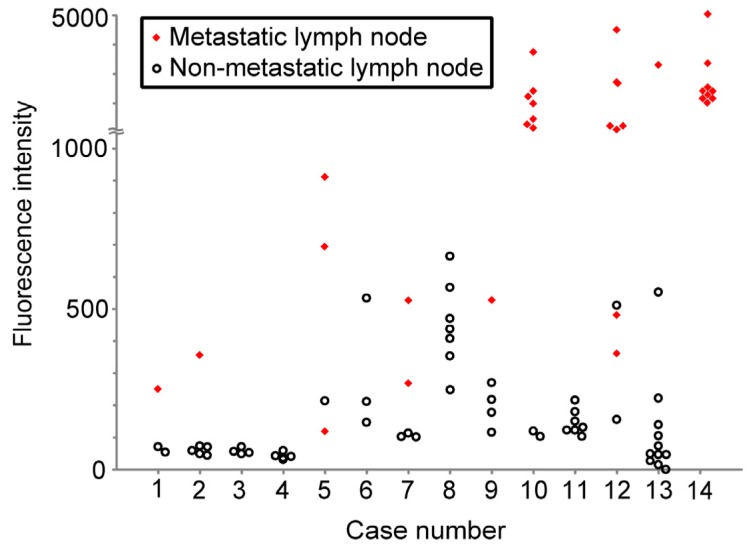
Scatter-plot for PpIX fluorescence intensities of metastatic and non-metastatic LNs. The signal intensities for the unmixed images of PpIX of LNs are plotted along the ordinate in each case. Closed diamonds denote metastatic LNs, opened circles denote non-metastatic LNs.

Figure 3 shows a scatter plot of the PpIX signal intensities acquired by spectral unmixing for metastatic and non-metastatic LNs. The mean PpIX signal intensity for metastatic LNs (1817 ± 1309 arb. units) was significantly increased compared with that for non-metastatic LNs (178 ± 168 arb. units) (unpaired Student’s *t* test, *p* < 0.0001)

**Figure 4:**
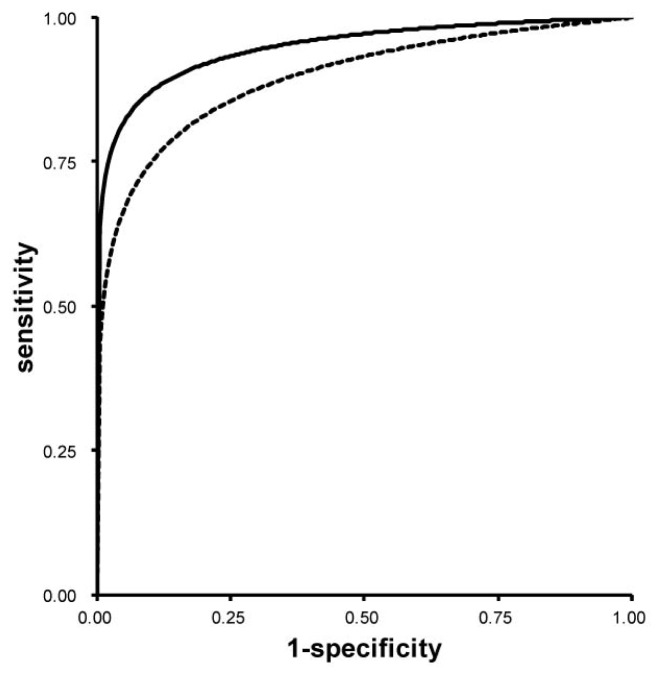
Fitted receiver-operating-characteristic (ROC) Curves. A ROC curve based on the PpIX fluorescence intensities analyzed by the spectral unmixing method is shown as a **bold line**. A ROC curve based on the PpIX fluorescence intensities analyzed by the spectral bandpass at the 640 nm is also depicted for comparison (**dotted line**).

To evaluate the efficacy of the diagnostic methods based on fluorescence intensity calculated by using the spectral unmixing, ROC curve analysis was performed (Figure 4). Fluorescence diagnosis of metastatic LNs was made on the basis of the fluorescence signal intensities. The ROC curve based on the PpIX fluorescence intensities analyzed by using the spectral bandpass at 640 nm is also shown to clarify the advantage of the spectral unmixing method in the LN examination. The sensitivity and the specificity, and the accuracy of the spectral unmixing method were 88.3%, 92.0%, and 87.4%, respectively. All values were acquired by calculation based on short distance from upper-left corner on the graph to each ROC curve as common optimal cut-off values. The measured values for the AUC of the spectral unmixing method and the spectral bandpass at 640 nm were 0.95 and 0.90, respectively (*p* = 0.009).

## 3. Discussion

In this study, we demonstrated that 5-ALA-induced PpIX fluorescence signal processed by using the spectral unmixing method is useful for the detection of CRC metastases in excised fresh human LNs. Application of this method may lead to intraoperative ultrarapid diagnosis of lymph node metastasis without preparation of histological sections. As far as we know, this is the first report on the application of 5-ALA-induced PpIX fluorescence to detect metastatic LNs in human CRCs.

A number of targeting fluorescent probes designed to visualize cancers have recently been developed [24–26]. Although many of them are valuable for identification of tumor cells in experimental animals, there are few probes applicable to a clinical setting now. 5-ALA-induced PpIX fluorescence has been shown to efficiently target human neoplasms, especially in the fields of urology and neurosurgery. Most recently, we have also shown the efficacy of 5-ALA-induced fluorescence in detecting metastatic gastric cancer in excised regional LNs by using color CCD camera [22]. Moreover, previous studies have indicated that 5-ALA can be administered in patients without serious side effect [15–18,27]. It is reported that the half-life of 5-ALA in the human body is approximately 45 min [28]. Indeed, 5-ALA was safely and tolerably applied to CRC patients with no apparent adverse reactions in this study.

Precise detection of PpIX fluorescence signals from metastatic cancer cells in human LNs was sometimes difficult because of spectral overlapping with strong autofluorescence emitted from collagenous tissues [23,29–32], unlike those emitted from paraaortic LNs of an orthotopic mouse model of human rectal cancer which we had previously studied [21]. The combination of optical bandpass filters and/or longpass filters has been used to remove such potentially interfering autofluorescence [21,22,33,34]. In this study, to specifically acquire PpIX fluorescence in metastatic LNs, we adopted the spectral unmixing method. As shown in Figures 2 and 4, PpIX fluorescence in LNs covered by tissue autofluorescence from collagen was successfully extracted, resulting in better performance in diagnosis of metastatic foci of LNs in CRC patients. In this study, the fluorescence intensity of PpIX was not apparently influenced by LN size and there was no influence of perinodal soft tissue on the PpIX fluorescence results. This is probably because we acquired cut-surface images of the LNs.

Recently, some genetic molecular techniques such as reverse transcription polymerase chain reaction have enabled diagnosis in clinical settings [35]. Such methods provide a very useful detection method of LN metastasis in cancer patients [36,37]. However, it is reported that LN samples excised at surgery were dissolved and lost during the procedure, making it impossible to perform subsequent analyses. On the other hand, observed specimens are preserved as is in our procedure, and PpIX fluorescence enables us to macroscopically identify metastasis in removed fresh LNs. Combination of the PpIX fluorescence gross imaging and the histological examination is one possible application for accurate diagnosis of LN metastases.

There are limitations in this study. Firstly, there were cases observed where the lymphoid follicles in excised LNs had non-specific PpIX fluorescence. This result was consistent with our previous data on regional LNs of gastric cancer patients [22]. In this study, we did not take into account the characteristic follicular fluorescence pattern for the evaluation of diagnostic performance of this method. With addition of the consideration of fluorescence patterns, the diagnostic performance of this method has potential for improvement. As we discussed in our previous report, the main reason for nonspecific deposition of PpIX is speculated to be due to inflammatory change [22]. Although the precise molecular mechanism of the nonspecific PpIX deposition has not been clearly determined, one possible reason for the increased PpIX deposition is relative deficiency of intracellular iron. In lymphoid follicles, administration of 5-ALA induces intracellular competition for available iron between processes of cell division and heme synthesis, resulting in PpIX deposition [38,39]. Through further analysis, we may be able to decrease the nonspecific PpIX deposition. Secondly, PpIX fluorescence of metastases less than 200 μm was too weak to detect under our system. Some recent studies have shown the prognostic significance of isolated tumor cells less than 200 μm in diameter in LNs of patients with stages I and II CRC [40,41]. In the present study, a LN indicated by a red diamond at the lowest position for case number 5 was not detected (Figure 3), because the cancerous focus was a metastasis of less than 200 μm. More sensitive imaging systems with improvement of light source would make it possible to increase the sensitivity. Thirdly, this study includes a relatively small number of cases. In our study, we confined to the cases in which tumor resection was completed 5–9 h after administration of 5-ALA, and no influence of operative time on the fluorescence signal intensity was apparent. However, large-scale study is needed to determine the ideal time between administration of 5-ALA and fluorescence imaging in the future. Furthermore, as there was only one case of undifferentiated colon cancer observed in our study, we could not examine the difference of PpIX fluorescence intensity between undifferentiated and differentiated colon cancers. As the incidence of undifferentiated colon cancer is generally lower than that of differentiated colon cancer [42], future large-scale study to analyze the relationship between PpIX fluorescence intensity and tumor grade is also necessary.

## 4. Methods

### 4.1. Patients

The single arm trial was performed at University Hospital, Kyoto Prefectural University of Medicine, between June 2009 and February 2011. The study protocol conformed to the ethical guidelines of the Declaration of Helsinki. All patients or their relatives provided written informed consent, and the Ethics Committee of Kyoto Prefectural University of Medicine approved all aspects of the study (C-403, C-671). The criteria for eligibility were preoperative histologically proven CRC that penetrates the muscle layer or invades the subserosa or pericolorectal connective tissue without visceral peritoneal coat (TNM classification by the International Union Against Cancer, >T2). Patients with the following clinical findings were excluded: acute porphyria, elevated liver enzymes, renal or hepatic insufficiency, intake of hypericin-containing medication, histamine H2 receptor antagonist or proton pump inhibitor, performance status (the Eastern Cooperative Oncology Group (ECOG) performance status, <2), pregnancy, and intestinal obstruction.

### 4.2. 5-ALA Administration

5-ALA hydrochloride (COSMO BIO Co., Ltd., Tokyo, Japan) dissolved in 20 mL of 50% glucose was applied orally at a concentration of 15 mg/kg of body weight at 2 h before surgery. Patients were protected from direct sunlight for 24 h after administration of 5-ALA. Fluorescence study was confined to the cases in which tumor resection was completed 5–9 h after administration of 5-ALA, and data from patients in whom resection was not completed 5–9 h after administration of 5-ALA were eliminated.

### 4.3. Tissue Processing

Immediately after LNs were operatively resected en bloc with primary tumors, LNs were isolated from the resected tissues and protected from light in sealed boxes. The LNs were then cut in half using stainless steel microtome blades. The cross-sectional fluorescence and white-light images of the half-cut LNs were obtained by using a macrozoom microscope. After the observations, the specimens were additionally stained with H & E and histopathologically diagnosed by pathologists without knowledge of the results of the fluorescence examinations.

### 4.4. Acquisition of Fluorescence Images and Reference Spectra for Spectral Unmixing

The system used for spectral unmixing was composed of a macrozoom fluorescence microscope (MVX10; Olympus, Tokyo, Japan) equipped with a monochrome charge coupled device (CCD) camera (ORCA-ER; Hamamatsu Photonics, Hamamatsu, Japan). Fluorescence mirror unit (U-MNV2; Olympus, Tokyo, Japan) combined with an excitation bandpass filter (BP400-410; Olympus, Tokyo, Japan), a barrier filter with cutoff of 455 nm (BA455; Olympus, Tokyo, Japan) and a dichroic mirror (DM455; Olympus, Tokyo, Japan) was attached to this fluorescence microscope. A mercury lamp was used for fluorescence excitation (USH1030; Olympus, Tokyo, Japan). Series of spectroscopic images were sequentially acquired by using a tunable filter (Varispec, CRi, Woburn, MA, USA) from 480 nm to 700 nm in 20-nm steps. Reference fluorescence spectra of 0.07 μM PpIX (Sigma-Aldrich, St. Louis, MO, USA) dissolved in dimethyl sulfoxide (DMSO) and collagen powder from bovine Achilles tendon, type I (Sigma-Aldrich, St. Louis, MO, USA) were also acquired by using the system. The maximum value of fluorescence intensity in the unmixed images of PpIX of LNs and that in the spectral bandpass images at 640 nm of LNs were used for the assessment of PpIX fluorescence in the specimens (Figures 3 and 4). White light images were also taken by using a color CCD digital camera (DP71; Olympus, Tokyo, Japan) for shape delineation.

### 4.5. Spectral Unmixing

Spectral unmixing is a method of resolving crosstalks among emission spectra from several materials. The method assumes that the signal intensity of each pixel in an acquired image can be expressed by linear combination of emission spectra from several materials that have known spectra [43]. The following algorithm of the variables for the explanation of the spectral unmixing methods is used: A three-dimensional data set of tissues with dimension *N_x_* × *N_y_* × *N_λ_* is collected with the macrozoom fluorescence microscope and the tunable filter, where *N_x_* and *N_y_* denote the number of pixels in the *x* and *y* directions and *N_λ_* denotes the data points of the fluorescence spectrum. The three-dimensional data set is reshaped to be a two-dimensional matrix of dimension *N_x_N_y_* × *N_λ_* to apply spectrum unmixing algorithm. We assumed that the two-dimensional data set *X* can be explained with the product of the concentration data set *C* of *N_x_N_y_* × *p* and the user-defined spectral data set *S* of *p* × *N_λ_* including the fluorescence spectrum of PpIX, where *p* as the number of user-defined spectra for the spectral unmixing,

(1) X=CS

The concentration matrix can be calculated as follows,

(2) $$C={\mathrm{XS}}^{t}{\left({\mathrm{SS}}^{t}\right)}^{-1}$$

The calculated concentration matrix composes the unmixed concentration map of PpIX, and thus we can precisely estimate the spatial distribution of PpIX without background contributions.

In this paper, fluorescence spectra of pure chemicals of PpIX and collagen type I were used as the user-defined spectra for spectral unmixing. The distribution of PpIX in LNs was estimated by plotting the score of the pure chemical of PpIX in the calculated concentration matrix.

### 4.6. Statistical Analysis

Statistical analysis was performed with commercial software (Microsoft Excel; Microsoft Corp., Redmond, WA, USA). Unpaired Student’s *t* test was used to assess the difference of the fluorescence intensity between metastatic and nonmetastatic LNs. Receiver-operating-characteristic (ROC) curves for the imaging method were generated by ROCKIT (version 0.9β) and ROCPLOT (version 1.0) software [44]. The area under the curve (AUC) was used as an index in evaluating the inherent capacity of a method to discriminate between “positive” and “negative” LNs.

## 5. Conclusions

This study showed that fluorescence diagnosis with 5-ALA is effective for detection of CRC metastases in excised fresh human LNs, suggesting a novel clinical approach to evaluate staging classification of human CRC. Although further improvements based on larger population studies are needed, this rapid and feasible method may be applicable to gross evaluation of resected LN samples in pathology laboratories.

## Figures and Tables

**Table 1:** Clinicopathological characteristics of enrolled patients and examined lymph nodes.

Case number	Age (years)	Sex	Histological grade	Tumor location	Tumor depth (T)	Nodal status (N)	Stage	Number of examined nodes
1	81	Female	Diff.	A	T4a	N2b	IIIC	3
2	75	Female	Diff.	A	T3	N1a	IIIB	6
3	74	Female	Diff.	S	T4a	N0	IIB	4
4	70	Male	Diff.	S	T2	N0	I	4
5	65	Male	Diff.	A	T3	N1b	IIIB	4
6	63	Male	Diff.	A	T3	N0	IIA	3
7	67	Male	Diff.	D	T4a	N1b	IIIB	5
8	69	Male	Diff.	S	T2	N0	I	7
9	66	Male	Diff.	R	T3	N1a	IIIB	5
10	46	Male	Diff.	R	T3	N2b	IIIC	9
11	72	Male	Diff.	C	T3	N0	IIA	7
12	61	Female	Diff.	S	T3	N2a	IIIB	9
13	83	Female	Undiff.	S	T4a	N1b	IIIB	12
14	56	Male	Diff.	S	T3	N2b	IIIC	9

Abbreviations: Diff., Differentiated; Undiff., Undifferentiated; A, Ascending colon; C, Cecum; R, Rectum; D, Descending colon; S, Sigmoid colon; Classified according to the 7th edition of UICC TNM classification for CRC.
